# *Notes from the Field*: Hepatitis C Transmission from Inappropriate Reuse of Saline Flush Syringes for Multiple Patients in an Acute Care General Hospital — Texas, 2015

**DOI:** 10.15585/mmwr.mm6609a4

**Published:** 2017-03-10

**Authors:** Sandi Arnold, Sharon K. Melville, Bonnie Morehead, Gilberto Vaughan, Anne Moorman, Matthew B. Crist

**Affiliations:** ^1^Texas Department of State Health Services; ^2^Texas Department of State Health Services, Health Service Region 7; ^3^Division of Viral Hepatitis, National Center for HIV/AIDS, Viral Hepatitis, STD, and TB Prevention, CDC; ^4^Division of Healthcare Quality Promotion, National Center for Emerging and Zoonotic Infectious Diseases, CDC.

In October 2015, the Texas Department of State Health Services (DSHS) was notified that a hospital telemetry unit nurse had been reusing saline flush prefilled syringes in the intravenous (IV) lines of multiple patients, a risk factor for patient-to-patient transmission of bloodborne pathogens (*1*).* This practice was discovered through an investigation undertaken by the hospital after the nurse was observed to frequently leave a partially filled syringe near a computer work station. State, regional, and local health departments, with consultation from CDC, collaborated with the hospital to investigate infection prevention lapses, assess risk to patients, perform patient notification, and provide bloodborne pathogen testing.^†^

Upon interview, the nurse reported reusing syringes during the previous 6 months, erroneously believing that this was a safe, cost-saving measure if no fluids were withdrawn into the syringe before injection of the saline flush (*1*,*2*). The nurse had been working in this unit for 18 months, had not worked at another health care facility before or during employment at the hospital, and reported that this practice was not taught by the hospital. The hospital voluntarily notified patients and offered bloodborne pathogen screening to patients who might have been cared for by the nurse during employment from April 2014 to October 2015, when the practice was recognized and corrected (*3*). Because all telemetry unit patients were required to have IV access, all patients cared for on the unit during shifts worked by the nurse were included in the notification.

During October 2015, notification letters were sent to patients via both certified and registered mail to inform them of a possible bloodborne pathogen exposure and a need for laboratory testing for Hepatitis B (HBV), Hepatitis C (HCV), and human immunodeficiency virus (HIV). The notification included locations where testing would be offered, a laboratory order form, and a 24-hour hospital hotline number for questions and concerns. The hospital provided testing free of charge through a commercial laboratory that coordinated testing at many satellite locations. Recommended laboratory testing consisted of a baseline screening test and a follow-up test at 6 months after the last potential exposure; exposure was defined as the last time a patient was on the telemetry unit while the nurse was working.^§^

Patients who did not have bloodborne pathogen testing or whose letter had been returned as undeliverable, and who had valid contact telephone information were telephoned individually by hospital staff members to provide notification, encourage testing, and request a current mailing address. Notification materials were re-sent to contacted patients; for those who could not be reached, additional address investigation was performed by DSHS using a search of state databases. As of October 2016, among 392 potentially exposed living patients, 262 (67%) had completed initial screening, and 182 (46%) had completed all recommended testing.

Among the 262 patients tested at least once for HBV, HCV, and HIV, four patients with newly diagnosed bloodborne pathogen infections were identified: two with HBV and two with HCV. A patient with known preexisting chronic HCV infection (patient A) had been hospitalized on the telemetry unit on the same day as patient B, one of the patients with newly diagnosed HCV. The second patient with newly diagnosed HCV infection did not share overlapping hospital days with any patient with known HCV infection, and the two patients with newly diagnosed HBV infection did not share overlapping hospital days with each other or any patient with a known HBV infection. Thus, no further epidemiologic evidence was identified that linked these three patients with newly diagnosed infections to a potential source patient. 

Specimens from patients A and B were sent to the laboratory in CDC’s Division of Viral Hepatitis for genotyping and molecular sequencing. Both patients were infected with HCV genotype 4a, which represents approximately 1% of all infections in the United States. Quasispecies (HCV intra-genotype variants) analysis was performed, and <0.38% nucleotide variation among intrahost HCV sequences from these two patients was detected (Figure). This result indicates transmission linkage between these two patients (*4*). Further epidemiologic investigation indicated that it was unlikely that these two patients had any contact outside the facility.

**FIGURE:**
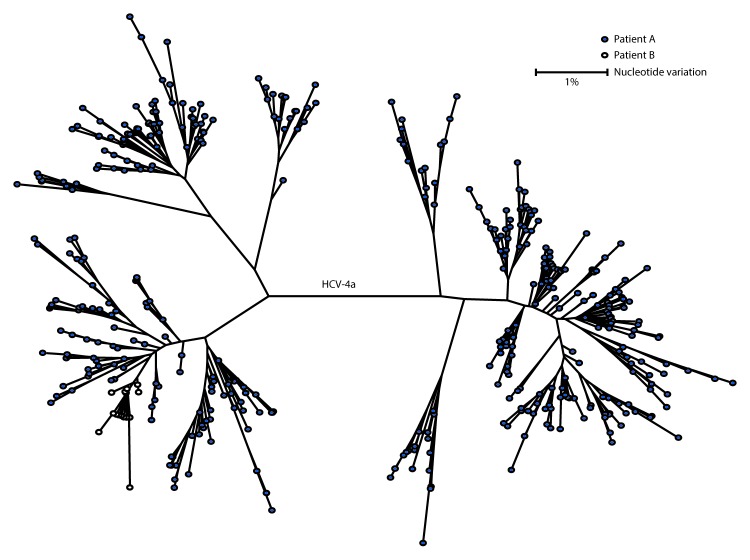
Genotyping and molecular sequencing* of hepatitis C virus^†^ specimens from two patients^§^ in an acute care general hospital — Texas, 2015 **Abbreviations:** HCV = hepatitis C virus; bp = base pairs. * Both patients were infected with genotype 4a and quasispecies (HCV intra-genotype variants) analysis demonstrates maximum nucleotide identity of 99.62% among intra-host HCV sequences. ^†^ E1-HVR1 region, 264 bp in length, only NGS454 unique sequences are shown. ^§^ Solid dots represent the quasispecies from the patient with known chronic HCV infection (patient A), and open dots represent quasispecies from the patient with newly diagnosed HCV infection (patient B). Although the viral variants are not identical between the two cases, the genetic distances in nucleotide variation between the cases are well below the threshold for defining transmission linkage.

Taken together, these findings indicate that at least one HCV infection was likely transmitted in the telemetry unit as a result of inappropriate reuse and sharing of saline flush syringes for multiple patients. This investigation illustrates a need for ongoing education and oversight of health care providers regarding safe injection practices. Hospitals and other settings where injections are prepared and administered should perform routine audits (*1*–*3*). Syringe reuse, if identified, should be immediately corrected and patient notification should be included as part of the institutional response (*1*–*3*).

## Footnotes

*
https://www.cdc.gov/injectionsafety/unsafepractices.html .

†
https://www.cdc.gov/hepatitis/outbreaks/healthcareinvestigationguide.htm .

§
https://www.cdc.gov/hepatitis/outbreaks/toolkit.htm .
